# The effect of photodynamic therapy and polymer solution containing nano-particles of Ag /ZnO on push-out bond strength of the sealers AH-Plus and MTA Fillapex

**DOI:** 10.4317/jced.54069

**Published:** 2017-09-01

**Authors:** Hamidreza Yavari, Negin Ghasemi, Baharak Divband, Yashar Rezaei, Golchin Jabbari, Salar Payahoo

**Affiliations:** 1 DDS, MSc, Associate Professor, Department of Endodontics, Dental Faculty, Tabriz University (Medical Sciences), Tabriz, Iran; 2 DDS, MSc, Assistant Professor, Department of Endodontics, Dental and Periodontal Research Center, Dental Faculty, Tabriz University (Medical Sciences), Tabriz, Iran; 3 Assistant Professor, Department of Chemistry, Tabriz University, Tabriz, Iran; 4 Assistant Professor, Department of Dental Biomaterials, Faculty of Dentistry, Tabriz University of Medical Sciences, Tabriz, Iran; 5 DDS, Endodontic Department, Dental and Periodontal Research Center, Faculty of Dentistry, Tabriz University of Medical Sciences, Tabriz, Iran; 6 DDS, Department of Oral and maxillofacial radiology, Dental and Periodontal Research Center, Faculty of Dentistry, Tabriz University of Medical Sciences, Tabriz, Iran

## Abstract

**Background:**

The aim of this *in vitro*study was to examine and compare the effect of photodynamic therapy and solution containing nano particles Ag/ZnO on adhesion of endodontic sealers to dentinal walls of human root canal.

**Material and Methods:**

Ninty single-rooted human teeth were selected and their clinical crown was cut from the cemento-enamel junction zone. Canals were prepared by RaCe rotary system and the smear layer was removed using 17% EDTA and 5.25% NaOCl. Samples were randomly divided into two groups of AH Plus and MTA Fillapex based on the sealer type and each group based on antimicrobial method was divided into two sub-groups of photodynamic therapy and polymer containing nano particles of Ag / ZnO and a control sub-group (N = 15). After obturation of canals with gutta-percha and sealers mentioned, the samples were incubated for a week at a humidity of 95% and 37°C and then 2mm thick discs were prepared from the middle region of roots for Push-out test. The maximum failure force was recorded in newton and converted to MPa. Then, 3 random specimens of each subgroup were evaluated by scanning electron microscopy. Statistical analysis was performed by Two way ANOVA and (*P*<0.05) was considered significant.

**Results:**

The highest (4.84 ± 1.63) and lowest (0.16 ± 0.075) push-out bond strength values were obtained in (AH Plus-PDT) and (MTA Fillapex-Ag/ZnO) respectively. Independent of antimicrobial method, AH Plus bond strength was significantly higher than the MTA Fillapex (*p*<0.001). For both sealers, PDT increased the bond strength significantly (*p*<0.001).

**Conclusions:**

Photodynamic therapy has a positive effect on the bond strength of AH Plus and MTA Fillapex sealers.

** Key words:**AH Plus, MTA Fillapex, Nano particles, Photodynamic therapy, Push-out.

## Introduction

Obturation of root canal system is an essential part of success in endodontics as it aims to close the leakage pathways from the coronal and apical directions(1). The most common material used for root canal obturation is gutta-percha with sealer. An ideal sealer should completely cover the canal space and adhere to the root canal wall and to the gutta-percha (2). Higher bond strength of sealer decreases leakage and also improves the stability of root canal filling materials (3). Based on research, adhesion of sealers to dentin is influenced by several factors including: chemical and physical characteristics of the sealer and its reaction with dentin, irrigation method and the smear layer removal (4-9).

The main purpose of root canal treatment is to remove microorganisms and their byproducts from root canal system and to prevent re-infection (10). One of the new methods introduced for canal disinfection is the use of metal- or polymer-based nanoparticles. Nanoparticles (NPs) are insoluble particles measuring less than 100 nm (11) that, the high ratio of surface area to volume gives them unique biological, physical and chemical characteristics. Biocompatibility, broad-spectrum and long lasting antibacterial activity are some of their advantages (12,13). Recently, photodynamic therapy (PDT) has been used to eliminate microorganisms of the root canal system *in vitro* and *in vivo* (14). This method is able to eliminate cultivable bacteria species especially *Enterococcus faecalis* from the canal and also to reduce the occurrence of bacterial resistance (15).

AH Plus is a resin-based sealer with widespread use which is biocompatible and has acceptable physical properties. In previous studies, the positive impacts of triple antibiotic paste (16), removing the smear layer (8,17), activating irrigation solution with ultrasonic(18), applying Er:YAG laser (7) on the bond strength of this sealer have been reported. Recently, the sealers with MTA base were introduced to achieve good biological characteristics of MTA. MTA Fillapex is one of these Sealers which is bactericidal and biocompatible (19). The positive impact of the smear layer removal (8) and relative humidity of dentin (6) on bond strength of this sealer have been shown.

Several studies have been conducted on the antimicrobial effectiveness of NPs and PDT but their impact on the dentin surface and adhesion of sealers has been less attended. The aim of this study was to evaluate the impact of these two methods on the adhesion of two types of resin-based and bioceramic sealers using the Push out test.

## Material and Methods

-Synthesis of Ag/ZnO nanoparticles

**Figure 1:**
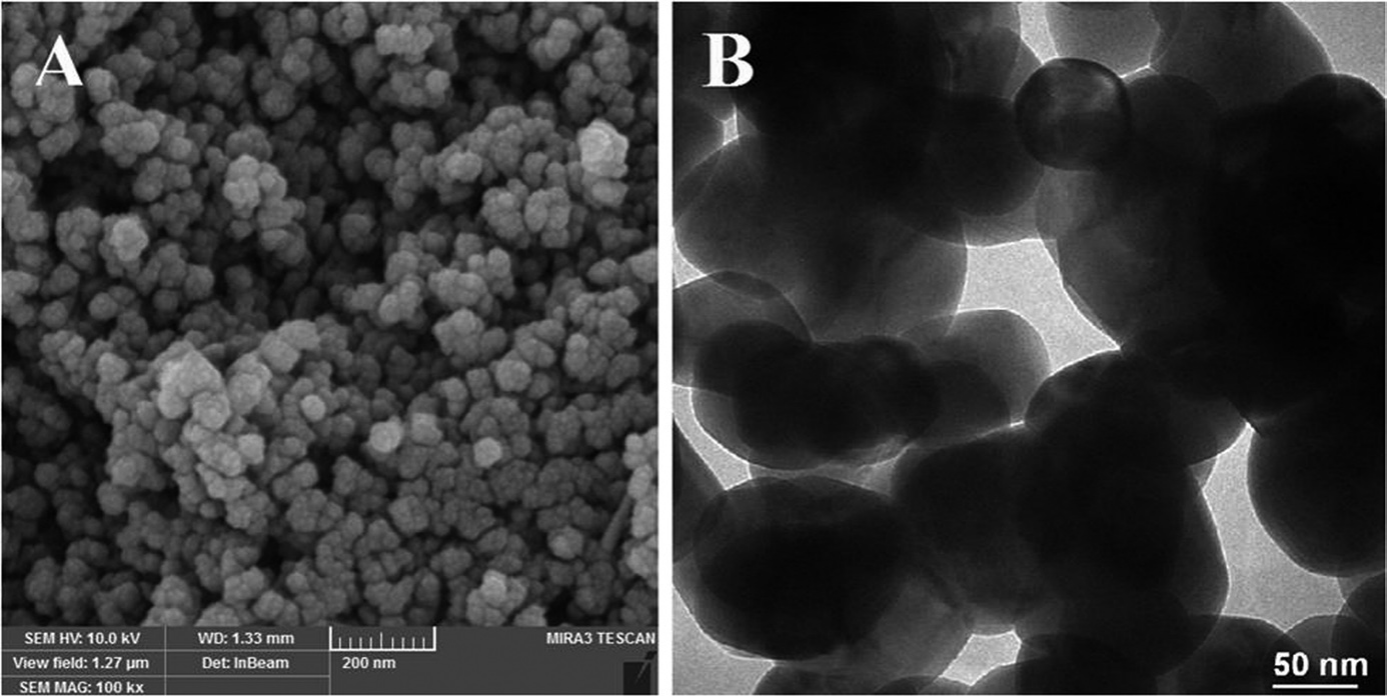
Photomicrographs of Ag/ZnO nanoparticles: A) SEM, B) TEM.

3.25g Zn(NO3)2 was dissolved in 15ml of ethanol and 40ml of distilled water. The mixture solution was moved to an ultrasonic bath and the aqueous solution of AgNO3 was dropped into the solution for 90 minutes. The suspension was transferred to a 200 mL teﬂon coated autoclave and kept at 120º C for 8 h. After the hydrothermal reaction, the precipitate separated from solution by centrifugation at 2000 rpm for 15 min and washed with deionized water several times and dried at 100°C overnight for further characterization. The obtained nanoparticles were dispersed in a polymer solution and the polymerization took place by increasing the temperature. Synthesized Ag/ZnO nanoparticles were characterized by Scanning electron microscopy MRIA3-FEG-SEM ( Tescan, Brno, Czech) and Transmission electron microscopy (Zeiss LEO 912 Omega) (Fig. 1).

-Tooth selection and preparation

90 extracted single-canal, closed apex, non-calcified and without curvature human maxillary central incisors teeth (due to perio-dontal problems) were selected. The removal of the calculus and soft tissue from root surfaces was done by ultrasonic device (Cavitron , Dentsply Ltd,Weybridge, UK).Samples were stored in a 0.5% chloramine-T solution until the time of the study. The crowns were sectioned from Cemento-enamel junction level using a diamond disc (SP 1600 Microtome, Leica, NuBlock, Germa-ny ). The working length (WL) was determined by using # 15 K-flexofile (Dentsply Mailefer, Ballaigue, Switzerland) at 1mm shorter than the apical foramen.Canal preparation was done with RaCe rotary system (FKG Dentaire, La-Chaux de Fonds, Switzerland) as follows: # 40/ 0.10 for the coronal third, # 35/ 0.08 for the middle third and # 30/0.06 up to WL. Normal saline was used during instrumentation and finally smear layer was removed using NaOCl (5.25%, 3 minutes) and EDTA (17%, 3 minutes).Then samples were randomly divided into two groups based on the sealer type and each group based on the antimicrobial method used were divided into two experimental sub-groups and one control group. In groups of disinfection with NPs, at the end of preparation 2 mL of polymer containing Ag / ZnO was used as a final rinse for 5 minutes.

In groups of PDT, this method was used as the last step of disinfection. In a dark room the canals were filled with a filter -sterilized solution of toluidine blue (2% aq.solution) for 5 minutes. Then light was applied into canals by using a diode laser (Handy laser sprint dental, RJ-laser, Winden, Germany). The wavelength of device was 638nm and output power was 150 mW. According to the manufacturer instruction light was applied as follows: 2.5 minutes of irradiation, 2.5 minutes of stop and 2.5 minutes of re-radiation to reactive free radicals without increasing the destructive temperature. For a 360-degree uniform radiation, a flexible plastic optical fiber (PACT, Cumdente, Tübingen, Germany) with a diameter of 200 micrometers and taper of 0.03 with cylindrical distributors were used. By placing this fiber in 3mm of working lenght, guiding light to the entire length of the canal and to the inaccessible areas such as fins and branches were ensured. After irradiation, canals were rinsed with 10 ml of normal saline.

In the control groups no adjunct method was used. Finally, the root canals were dried with paper points and obturated with gutta-percha and AH Plus (Dentsply, Dentrey, Kostanz, Germany) and MTA Fill apex (Angelus, Londrina, PR, Brazil) sealer using lateral condensation technique. Samples were stored at 95% humidity and 37°C for a week and then 2mm-thick dental disks from the middle third were prepared using a diamond disc with water coolant.

-Push-out bond strength test

**Figure 2:**
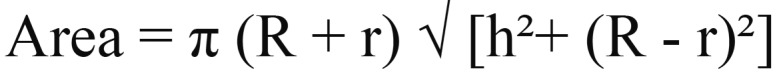
Formula.

Push-out testing was conducted by Universal testing machine (Model H5K-S; Hounsfield Test Equipment, Surrey, England). The force was applied in apico-coronal direction to the samples by using a 1.1 mm diameter cylindrical plunger parallel to the long axis of the tooth and at crosshead tip speed of 1 mm / min. The Maximum force which caused the bond failure and displacement of filling material was recorded in Newton (N). Push-out bond strength is obtained in MPa by dividing the load in N by the area of the bonded interface. The bonded area of each section was calculated using the following formula: (Fig. 2).

Where R is the coronal radius and r is the apical radius of disc, h is disc thickness and π=3.14.

-Preparations for SEM evaluating 

3 specimens of each group were randomly selected to scan with a scanning electron microscope MRIA3-FEG-SEM ( Tescan, Brno, Czech), to investigate the bond failure mode. Longitudinal grooves were created at two surfaces of the dental disks symmetrically by a diamond disc without entering the canal space and two pieces were separated by a hammer and chisel. Then the sections produced were coated by gold as a conductor of electric current and scanning electron micrographs were prepared.

-Statistical analysis

Data analysis by kolmogorov-smirnov test showed normal distribution of data. Two- way ANOVA analysis was used to assess the effect of the type of sealer and disinfection method on bond strength to dentin. Post hoc Tukey and independent t-tests were used for pair-wise comparisons of groups. Statistical significance was defined at *p* <0.05.

## Results

**Figure 3:**
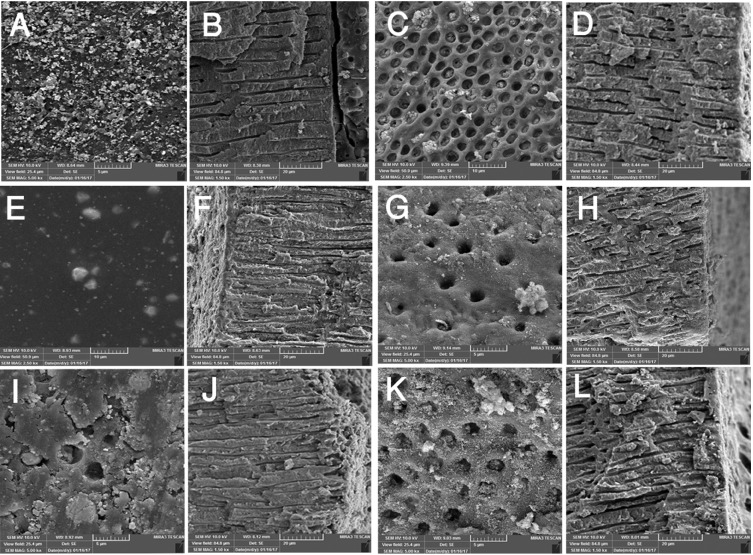
SEM photomicrogrographs of the studied groups. ( A and B) NPs and AH Plus, (C and D) NPs and MTA Fillapex, (E and F) PDT and AH Plus, (G and H) PDT and MTA Fillapex, (I and J) control and AH Plus, (K and L) control and MTA Fillapex.

The mean and standard deviation of bond strength for the studied groups have been shown in  Table 1. The highest and lowest mean push-out bond strength values were obtained in (AH Plus-PDT) and (MTA fillapex- Ag /ZnO) respectively. Regardless of antimicrobial method, AH Plus sealer had higher bond strength than MTA Fillapex in all groups (*p* <0.001). PDT method significantly increased the bond strength of both sealers (*p* <0.001). Nano disinfection method had significantly negative effect on the bond strength of AH Plus sealer (*P* <0.001), but for MTA Fillapex sealer the difference between the NPs and the control group was not significant (*p* = 0.071). In SEM evaluations, the failure patterns for AH Plus sealer were cohesive and for MTA Fillapex were mixed pattern (Fig. 3). Penetration of AH plus sealer into dentinal tubules was observed generally and deep penetration was observed in PDT group (Fig. 3F). Despite the presence of open dentinal tubules MTA fillapex did not penetrate into the most (Fig. 3C,G,K).

**Table 1:**
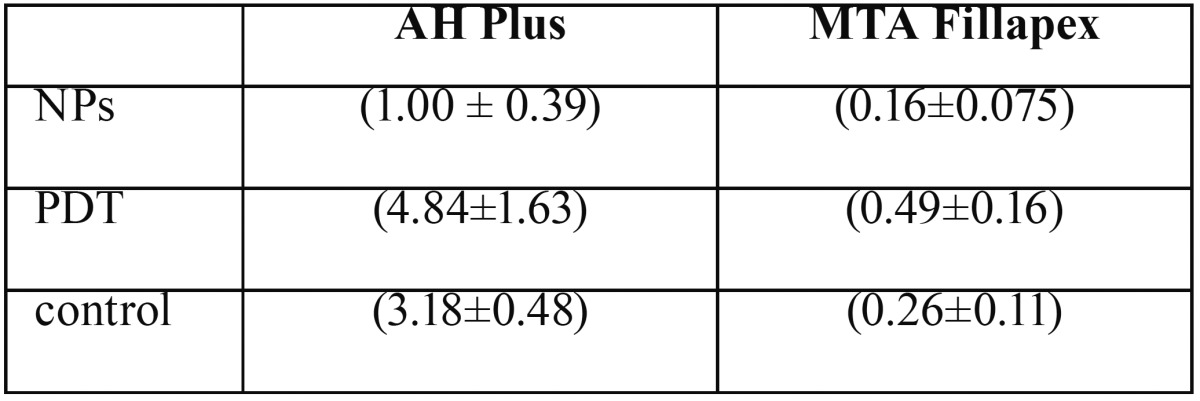
(Mean±SD) bonding strength of sealers to dentin.

## Discussion

This study has compared the bond strength of AH plus and MTA Fillapex sealers to the root canal dentin after using the final disinfection protocol including PDT and antibacterial NPs. PDT method unlike NPs , had a positive effect on the bond strength of both sealers.

Adhesion is one of the basic features for root canal sealers (3,20) and is essential to maintain the integrity of sealer-dentin interface during mechanical stresses applied due to occlusion forces and restorative processes such as post space preparation (21-23). Filling materials with lower bond strength have probably more defects at the interface of dentin-filling materials, and these defects can be paths for reinfection and endodontic treatment failure (24). The push-out test was used in this study to evaluate the adhesion of sealers to dentin, as it is effective and reliable (25) and better reflects the clinical conditions (26).

In this study, two different resin-based and calcium silicate-based sealers have been used and AH plus sealer showed greater adhesion to radicular dentin in comparison to MTA fillapex. AH Plus has been used as the gold standard of sealers and as the control group in previous studies (27). MTA Fillapex is one of the recently introduced calcium silicate-based sealers which was produced to have the favorable characteristics of the MTA, and for compensation of the features that makes MTA undesirable as a sealer such as working time, setting time and hard handling (19).

SEM images with high magnification allows for highly detailed observation of sealer adaptation and penetration into dentinal tubules (28) and the failure mode due to push out test can be investigated in details. In this study failure modes were cohesive for AH Plus sealer and mixed pattern for MTA Fillapex (Fig. 3F). The cohesive failure represents a strong bond between AH plus and radicular dentin which confirms the push-out test values. Sealer penetration into dentinal tubules and tag formation was observed generally. In MTA fillapex samples, dentinal tubules were visible in adhesive failure areas and although the orifices were open, sealer did not well penetrate all tubules (Fig. 3C,G,K).This pattern have been reported for other sealers previously (7,29).

Higher bond strength for AH Plus compared to MTA Fillapex has been reported in the litreture (6,30) and the results of our study was in consistence with those findings. However, Assmann *et al.* (31) reported similar resistance to dislodgement for both sealers. One of the differences between the two sealers is in the setting time, and MTA fillapex could not flow out, form the sealer tag and bond to the canal irregularities as a result of faster setting time. In addition, there is the possibility of formation of strong covalent bonds between exposed amino groups of dentin collagen and open epoxy rings in AH Plus sealer (27).

Disinfection of the root canal space is one of the main goals of root canal therapy and is necessary for healing of periapical diseases or for preventing them (32). PDT and antibacterial NPs are newly introduced root canal disinfectinion methods. Several studies have been conducted on the antimicrobial effectiveness of these two but their impact on the dentin surface and adhesion of sealers has been less attended in this studies

Ag nanoparticles prevent cell proliferation in the ionized form and cause cellular lysis (12), and ZnO nanoparticles destroy the bacteria by disrupting cell membranes (13). These particles together with (Ag / ZnO) have a higher antibacterial activity (33). PDT is a method in which by irradiating light with a specific wavelength to light-sensitive material (photosensitizer), localized in targeted tissue, become activated and produce free radicals which cause oxidative damage of target cells (34). The disadvantage in both methods is the reduced effectiveness in the presence of pulp tissue and root canal content (35), and therefore in this study they were used as an adjunct and final step in root canal disinfection process. Our study showed that NPs had a negative effect on AH plus adhesion to radicular dentin. The deposition of NPs on the radicular dentin surface may reduces the contact area and the adhesion of sealer to dentin. They also block the orifices of the dentinal tubules and prevent the sealer tag formation. Further studies are needed to investigate the effects of nanoparticles on the surface properties of dentin and their reaction with canal filling materials.

Conversely PDT showed positive effect on the adhesion of the both sealers in this study. Increased adhesion of resin-based sealers to dentin after treatment with laser Er:YAG (36) Nd: YAG (37) and the 980-nm diode laser (38) have been reported previously . The results of our study using diode laser for PDT also are consistent with these results. However Ok *et al.* (26) who had used the LED for PDT reported that it had no significant effect on bond strength of AH Plus sealer. The difference could be attributed to the light sources. Laser irradiation to the dentin surface removes the smear layer and also causes changes in the morphology of the dentin (39,40), thus, by increasing the irregularities and dentinal contact surface, the adhesion of sealer to dentin increases. These changes have been observed with an electron microscopy (39,40). SEM evaluation in the present study revealed deeper penetration of AH plus into the dentinal tubules with PDT using laser (Fig. 3F)

Tooth discoloration is an adverse effect of PDT when toluidine blue is used as photosensitizers. The alcohol compounds can be used to clean up this color material but given that a resin-based sealer was used in this study, no attempt was made to clean the dye because of the possibility of interference, which is of the limitations of this study. Studies should be continue to finding a way to benefit from the potential long term antibacterial effects of Ag/ZnO NPs despite the effect on push-out test observed in this study.

## Conclusions

Sealer adhesion to dentin is influenced by the type of sealer and canal disinfection method used. Considering the limitations of this study PDT is an appropriate adjunctive disinfection method for endodontic treatment because in addition to anti-bacterial property it can increase the adhesion of sealer to dentin.
